# Expression of DHA-Metabolizing Enzyme Alox15 is Regulated by Selective Histone Acetylation in Neuroblastoma Cells

**DOI:** 10.1007/s11064-017-2448-9

**Published:** 2017-12-12

**Authors:** Christabel Fung-Yih Ho, Claire Poh-Ee Bon, Yee-Kong Ng, Deron R. Herr, Jui-Sheng Wu, Teng-Nan Lin, Wei-Yi Ong

**Affiliations:** 1 Department of Anatomy, National University of Singapore, Singapore, 119260 Singapore; 2 Department of Pharmacology, National University of Singapore, Singapore, 119260 Singapore; 3 Graduate Institute of Life Sciences, National Defense Medical Center, Taipei, Taiwan; 4 Institute of Biomedical Sciences, Academia Sinica, Taipei, Taiwan; 5 Neurobiology and Ageing Research Programme, National University of Singapore, Singapore, 117456 Singapore

**Keywords:** Epigenetic effects, Histone acetylation, Alox15, DHA, Brain development, Resolvin D1, PUFA, Cerebral cortex

## Abstract

The omega-3 polyunsaturated fatty acid, docosahexaenoic acid (DHA) is enriched in neural membranes of the CNS, and recent studies have shown a role of DHA metabolism by 15-lipoxygenase-1 (Alox15) in prefrontal cortex resolvin D1 formation, hippocampo-prefrontal cortical long-term-potentiation, spatial working memory, and anti-nociception/anxiety. In this study, we elucidated epigenetic regulation of Alox15 via histone modifications in neuron-like cells. Treatment of undifferentiated SH-SY5Y human neuroblastoma cells with the histone deacetylase (HDAC) inhibitors trichostatin A (TSA) and sodium butyrate significantly increased Alox15 mRNA expression. Moreover, Alox15 expression was markedly upregulated by Class I HDAC inhibitors, MS-275 and depsipeptide. Co-treatment of undifferentiated SH-SY5Y cells with the p300 histone acetyltransferase (HAT) inhibitor C646 and TSA or sodium butyrate showed that p300 HAT inhibition modulated TSA or sodium butyrate-induced Alox15 upregulation. Differentiation of SH-SY5Y cells with retinoic acid resulted in increased neurite outgrowth and Alox15 mRNA expression, while co-treatment with the p300 HAT inhibitor C646 and retinoic acid modulated the increases, indicating a role of p300 HAT in differentiation-associated Alox15 upregulation. Increasing Alox15 expression was found in primary murine cortical neurons during development from 3 to 10 days-in-vitro, reaching high levels of expression by 10 days-in-vitro—when Alox15 was not further upregulated by HDAC inhibition. Together, results indicate regulation of Alox15 mRNA expression in neuroblastoma cells by histone modifications, and increasing Alox15 expression in differentiating neurons. It is possible that one of the environmental influences on the immature brain that can affect cognition and memory, may take the form of epigenetic effects on Alox15 and metabolites of DHA.

## Introduction

There has been much recent interest in the omega-3 polyunsaturated fatty acid (PUFA) docosahexaenoic acid (DHA) due to its reported anti-inflammatory [1] and neuroprotective roles [2], as well as effects on cognition and behavior [3, 4]. Many studies have also demonstrated beneficial effects of DHA in learning and memory [5–7]. Lipoxygenases are enzymes that act on PUFAs including DHA, to generate hydroxyperoxide products that act as signaling molecules or induce structural or physiological changes in the cell. DHA is the main *n-*3 fatty acid in neuronal membranes [8], and is metabolized by 15-lipoxygenase (Alox15) [9]. The latter produces pro- and anti-inflammatory mediators depending on substrates available. Arachidonic acid released from membrane glycerophospholipids by cytosolic phospholipase A_2_ (cPLA_2_) is metabolized by Alox15 to produce 15S-hydroxyeicosatetraenoic acid (15S-HETE) which has pro-inflammatory properties [10]. At the same time 15S-HETE is an endogenous ligand for peroxisome proliferator-activated receptor gamma (PPARγ), which has anti-inflammatory effects [11]. Alox15 can also metabolize DHA to produce resolvin D1 and neuroprotection D1, which have anti-inflammatory actions [12, 13]. Resolvin D1 modulates the infiltration of neutrophils [14] and reduces inflammation by inhibiting production of the pro-inflammatory cytokine, interleukin-1β (IL-1β) [1]. Recently, Alox15 has been found to have effects on synaptic plasticity. Antisense knockdown of the enzyme in the rat prefrontal cortex results in reduced levels of resolvin D1 and deficits in hippocampo-prefrontal cortical long-term-potentiation (LTP) and spatial working memory [15]. Alox15 inhibition in the prefrontal cortex reduces the anti-nociceptive effect of a DHA-rich nutraceutical, in a mouse model of neuropathic orofacial pain, suggesting a role of the enzyme in supraspinal antinociception [16]. At the spinal cord level, resolvin D1 reduces inflammatory pain in mice by normalizing aberrant spinal synaptic plasticity involved in pain hypersensitivity [17].

The 5′-flanking promoter region of the human Alox15 gene contains putative binding sites for transcription factors including signal transducer and activator of transcription 6 (STAT6), activator protein 2 (AP-2), GATA, nuclear factor 1 (NF-1), and P-1 [18]. Transcriptional activation of Alox15 by interleukin-4 (IL-4) has been reported - in monocytes [19], endothelial cells [18] and lung epithelial cells [20]. IL-4 induction of Alox15 expression occurs via the STAT6 pathway [21]. Activation of the cyclic GMP/protein kinase G pathway also induces Alox15 expression in human colon cancer cells [22]. Epigenetic modification of the Alox15 gene by histone deacetylases (HDACs) is an additional mechanism for regulating its expression [23, 24]. HDACs remove acetyl groups from lysine residues on histones, promoting a condensed and transcriptionally-inaccessible form of chromatin (heterochromatin). In contrast, histone acetyltransferases (HATs) add acetyl groups to histone lysine residues to promote a more accessible conformation of chromatin, and facilitate gene transcription.

To date, there is limited data regarding regulation of Alox15 in neuronal cells. In this study, we elucidate epigenetic regulation of Alox15 in SH-SY5Y human neuroblastoma cells using HDAC and HAT inhibitors. Results indicate expression of DHA-metabolizing enzyme Alox15 is regulated by selective histone acetylation in these cells.

## Materials and Methods

### Chemicals

All reagents were of analytical grade and used as received without further purification. Dimethyl sulfoxide (DMSO), trichostatin A (TSA), tubacin, C646 and retinoic acid (RA) were purchased from Sigma-Aldrich (St. Louis, MO, USA). Sodium butyrate (NaBT), MS-275 and butyrolactone-3 (MB-3) were purchased from Santa Cruz Biotechnology (Santa Cruz, California, USA). Depsipeptide was purchased from ApexBio (ApexBio, Texas, USA). NU9056 was purchased from Tocris Bioscience (Tocris Bioscience, Bristol, UK). TMP 195 was purchased from Axon Medchem (Axon Medchem, Groningen, Netherlands) and PD146176 was obtained from Cayman Chemical (Cayman Chemical, MI, USA). Stock solutions were prepared in DMSO and further diluted in cell culture medium for use. The equivalent concentration of DMSO was used as vehicle controls, with final concentrations of 0.1% or less, in cell culture media.

### Cell Cultures

Fully differentiated primary cortical neurons were prepared from E15.5 mouse embryos and cultured in Neurobasal plus B-27 medium (Gibco, NY, USA) containing 2 mM l-glutamine, 10 μM glutamate, 1.6% fetal bovine serum (FBS), 0.4% horse serum and penicillin/streptomycin. On days in vitro (DIV) 3, cells were treated with 1 µM cytosine arabinoside (Ara-C) for 72 h to prevent glial proliferation and maintained in serum-free Neurobasal plus B-27 medium at 37 °C in a humidified 5% CO_2_ incubator. Experiments were conducted on DIV 10–14 [25].

SH-SY5Y human neuroblastoma cells (CRL-2266) were obtained from American Type Culture Collection (ATCC^®^). SH-SY5Y cells were cultured in Dulbecco’s Modified Eagle’s Medium (DMEM) containing 10% heat-inactivated FBS and 1% penicillin/streptomycin (Gibco-Invitrogen, CA, USA). SH-SY5Y cells were maintained in 100 mm dishes (Greiner Bio-One, USA) and incubated under standard conditions of 37 °C and 5% CO_2_. They were regularly passaged using 25% Trypsin–EDTA (Gibco, NY, USA).

Differentiation of SH-SY5Y cells was carried out by seeding cells in 100 mm culture dishes and cell growth was constantly monitored up to approximately 50–60% confluency. RA was then added at a final concentration of 10 μM in DMEM with 10% FBS.

### Effect of HDAC and HAT Inhibitors on Alox15 mRNA Expression in Primary Murine Cortical Neurons

#### Treatment of Primary Cortical Neurons with Various HAT Inhibitors and TSA

TSA is a pan-HDAC inhibitor that blocks the activity of class I and II HDACs [26]. C646 is a HAT inhibitor that inhibits p300 HAT specifically [27], whereas NU9056 is a Tip60 HAT-specific inhibitor [28]. To examine the effects of HDAC and HAT inhibitors on Alox15 expression, primary murine neurons were co-treated with C646 or NU9056 and 0.5 μM TSA for 24 h. The neurons were treated as follows: (1) DMSO as vehicle control, (2) 0.5 µM TSA, (3) 20 μM C646 and 0.5 µM TSA, or (4) 20 μM NU9056 and 0.5 µM TSA.

### Effect of Alox15 Inhibitor, PD146176, on MAP-2, NeuN, α-Tubulin and Alox15 mRNA Expression in Primary Murine Cortical Neurons

#### Treatment of Primary Cortical Neurons with PD146176

Primary murine neurons were cultured and harvested at DIV 3, 5 and 10, and expression of neuronal markers MAP-2, NeuN and α-tubulin, as well as Alox15 determined at these times. To examine the effects of Alox15 on neuronal markers, primary murine neurons were treated with the specific Alox15 inhibitor, PD146176 (1 µM). Protein expression of MAP-2, NeuN, Alox15, α-tubulin and β-actin were determined by Western blots.

##### Semi-quantitative RT-PCR Analyses

Total RNA from primary murine neurons (1 μg) was incubated with 200 units of MMLV reverse transcriptase (RTase, Clontech; Palo Alto, CA) in a buffer containing a final concentration of 50 mM Tris–Cl (pH 8.3), 75 mM KCl, 3 mM MgCl_2_, 20 units of RNase inhibitor, 1 μM polydT oligomer, and 0.5 mM of each dNTP in a final volume of 20 μl. The reaction mixture was incubated at 42 °C for 1 h and then 94 °C for 5 min to inactive the enzyme. A total of 80 μl DEPC treated water was added to the reaction mixture before storage at − 70 °C. 5 μl of the RT reaction solution was used in the PCR reaction. PCR was carried out in a 50 μl final volume containing 200 μM each of dATP, dCTP, dGTP, and dTTP, 5 pmole of each primer, 1.25 units of Tag polymerase (BRL; Gaithersburg, MD), 20 mM Tris–Cl (pH 8.4), 1.5 mM MgCl_2_, and 50 mM KCl. The mixture was incubated in a thermal cycler for 35 cycles using the following profile: 94 °C for 7 min, then repeat cycles of 94 °C for 45 s; 55 °C for 45 s; 72 °C for 90 s. Samples were then incubated at 72 °C for 7 min and cooled to 4 °C (GeneAmp 2400, PE; Norwalk, CT). PCR products were run on 2% agarose gel for DNA fragment size verification. Primers used were: 5′-CAGGGATCGGAGTACACGTT and 5′-GATTGTGCCATCCTTCCAGT for Alox15 [29]; and 5′-TCCCTCAAGATTGTCAG and 5′-AGATCCACAACGGATAC for GAPDH. Statistical differences were analyzed using one-way ANOVA with post-hoc Fisher’s protected *t* test, where *p* < 0.05 was considered significant.

##### Western Blot Analyses

Western blot analysis of proteins was performed as previously described [30], with mouse monoclonal antibody to Alox15 (1:500, Abcam, New Territories, HK) and antibodies to MAP-2, NeuN or α-tubulin (1:500, Santa Cruz, CA, USA). Protein bands were visualized by an enhanced chemiluminescence system (Merck Millipore, MA, USA). Statistical differences were analyzed using one-way ANOVA with post-hoc Fisher’s protected *t* test, or Student’s *t* test, where *p* < 0.05 was considered significant.

### Effect of HDAC and/or HAT Inhibitors on SH-SY5Y Cells

#### Experiments on Undifferentiated SH-SY5Y Cells

##### Treatment of Undifferentiated SH-SY5Y Cells with Histone Deacetylase (HDAC) Inhibitors

Undifferentiated SH-SY5Y cells were treated with general HDAC inhibitors TSA (0.5 µM) and sodium butyrate (5 mM), and specific HDAC inhibitors, MS-275 (5 µM) and depsipeptide (10 nM), for 24 h. Sodium butyrate is a general HDAC inhibitor that blocks the activity of class I and II HDACs [31] and is effective in the millimolar range [32]. MS-275 inhibits HDACs 1, 2 and 3 activities, and depsipeptide is an inhibitor of HDACs 1 and 2 [33]. MS-275 inhibits HDAC activity at the micromolar range [34, 35] and depsipeptide is effective at the nanomolar range [24]. TMP 195 is a Class IIa specific HDAC, and is effective in the nanomolar range [36]. Each group consisted of four biological replicates.

##### Treatment of Undifferentiated SH-SY5Y Cells with HAT Inhibitors and TSA

To investigate the effect of HATs on TSA-induced Alox15 expression, SH-SY5Y cells were treated individually or in combination with 0.5 μM TSA using inhibitors from three different classes of HATs: GCNS5 HAT (MB-3, 20 µM), p300 HAT (C646, 20 µM) and Tip60 HAT (NU9056, 20 µM). The treatments were carried out in four groups: (1) DMSO as vehicle control, (2) 0.5 µM TSA, (3) HAT inhibitor, and (4) HAT inhibitor and 0.5 µM TSA. Cells were co-treated with HAT inhibitors and TSA or vehicle for 24 h.

##### Treatment of Undifferentiated SH-SY5Y Cells with HAT Inhibitors and Sodium Butyrate

To investigate the effect of HATs on sodium butyrate-induced Alox15 expression, SH-SY5Y cells were treated individually or in combination with sodium butyrate using inhibitors of p300 HAT (C646, 20 µM) or Tip60 HAT (NU9056, 20 µM). The treatments were carried out in four groups: (1) DMSO as vehicle control, (2) 5 mM sodium butyrate, (3) HAT inhibitor, and (4) HAT inhibitor and 5 mM sodium butyrate. Cells were co-treated with HAT inhibitors and sodium butyrate or vehicle for 24 h.

##### Treatment of Undifferentiated SH-SY5Y Cells with HAT Inhibitors and MS-275

To investigate the effect of HATs on MS-275-induced Alox15 expression, SH-SY5Y cells were treated individually or in combination with MS-275 using inhibitors of p300 HAT (C646, 20 µM) or Tip60 HAT (NU9056, 20 µM). The treatments were carried out in four groups: (1) DMSO as vehicle control, (2) 5 μM MS-275, (3) HAT inhibitor, and (4) HAT inhibitor and 5 μM MS-275. Cells were co-treated with HAT inhibitors and MS-275 or vehicle for 24 h.

##### Treatment of Undifferentiated SH-SY5Y Cells with HAT Inhibitors and Depsipeptide

To investigate the effect of HATs on depsipeptide-induced Alox15 expression, SH-SY5Y cells were treated individually or in combination with depsipeptide using inhibitors of p300 HAT (C646, 20 µM) or Tip60 HAT (NU9056, 20 µM). The treatments were carried out in four groups: (1) DMSO as vehicle control, (2) 10 nM depsipeptide, (3) HAT inhibitor, and (4) HAT inhibitor and 10 nM depsipeptide. Cells were co-treated with HAT inhibitors and depsipeptide or vehicle for 24 h.

#### Experiments on Retinoic Acid Treated SH-SY5Y Cells

##### Treatment of SH-SY5Y Cells with Retinoic Acid and HAT Inhibitors

To examine the effects of HAT inhibitors on Alox15 expression during RA-induced differentiation of SH-SY5Y cells, undifferentiated SH-SY5Y cells were incubated with RA and C646 or NU9056 for 72 h. SH-SY5Y cells were treated as follows: (1) DMSO as vehicle control, (2) 20 μM HAT inhibitor, (3) 10 μM RA, and (4) 10 μM RA and 20 μM HAT inhibitor. The cells were harvested after 72 h and RNA extracted and analyzed by RT-PCR.

##### Quantification of Neurite Lengths of SH-SY5Y Cells

Images of SH-SY5Y cells were captured using the EVOS FL Cell Imaging System at ×20 magnification, and neurite lengths quantified using the ImageJ Simple Neurite Tracer software plugin, by tracing observable neurites extending from cell bodies in the images [37]. An overall mean neurite length (µm) of 350 cells was obtained for each treatment group. Possible differences between treatment groups were analyzed using two tailed t test and one-way ANOVA with Bonferroni’s multiple comparison post-hoc test, where *p* < 0.05 was considered significant.

### Real-Time RT-PCR

Total RNA from SH-SY5Y cells was extracted with the RNeasy Mini kit (Qiagen, Hamburg, Germany). Reverse transcription of RNA to complementary DNA (cDNA) was performed as previously described [38]. The cDNA obtained was quantified by real-time PCR (RT-PCR) using the TaqMan^®^ Universal PCR Master Mix (Applied Biosystems, CA, USA), with TaqMan^®^ Gene Expression Assay Probes for Alox15 (Hs00609608_m1), NSE (Hs00157360_m1), β-III-tubulin (Hs00801390_s1), and β-actin (#4326315E) (Applied Biosystems, CA, USA). Real-time PCR was performed and the relative fold changes were quantified using the 2^−ΔΔCT^ method as previously described [38]. All reactions were performed in triplicates and the mean and standard error calculated. Statistical differences were analyzed using two tailed t test and one-way ANOVA with Bonferroni’s multiple comparison post-hoc test, where *p* < 0.05 was considered significant.

### Immunocytochemistry

SH-SY5Y cells were plated at a density of 2 × 10^5^ cells on poly-l-lysine-coated coverslips placed in 24-well plates and grown to 80% confluency before administration of treatment. After treatment, the cells were fixed before antigen retrieval and permeabilization. They were then blocked in 1% BSA and incubated overnight at 4 °C with Alox15 specific antibody (ab119774, 1:50 in blocking buffer, Abcam, New Territories, HK), followed by secondary incubation with anti-mouse Alexa Fluor 488 (Applied Biosystems, CA, USA; diluted 1:100 in 1% BSA) for 1 h at room temperature. Nuclei were labelled and coverslips mounted with the Prolong Gold Antifade Mountant DAPI (Invitrogen, USA). Samples were analyzed and images captured using a confocal microscope (Zeiss, OR, USA).

### Quantitative Image Analysis

The fluorescence intensity of cells were measured as previously described [38]. The net fluorescence intensity was calculated for each image according to the following formula: Corrected total cell fluorescence (CTCF) = Integrated Density − (Area of selected cell × Mean fluorescence of background readings). The mean and standard error were calculated, and significant differences were analyzed using one-way ANOVA with Bonferroni’s multiple comparison post-hoc test, where *p* < 0.05 was considered significant.

### Lactate Dehydrogenase (LDH) Assay

SH-SY5Y cells were plated at a density of 2 × 10^5^ cells in 24-well plates, and grown to 80% confluency before administration of treatment. The specific Alox15 inhibitor, PD146176, was used for the selective inhibition of Alox15 activity. After 24 h, cell viability was assessed by colorimetric determination of lactate dehydrogenase (LDH) release, using the LDH Cytotoxicity Detection Kit (Roche, Mannheim, Germany). The plate was read at an excitation wavelength of 490 nm on the Tecan Infinite^®^ 200 microplate reader (Tecan Group Ltd., Maennedorf, Switzerland). Percentage cytotoxicity was calculated according to the formula:

$${\text{Cytotoxicity}}\left( \% \right)=\frac{{{\text{Experimental value-low control}}}}{{{\text{High control-low control}}}} \times {\text{100}}$$

The mean cytotoxicity values were then normalized against vehicle-treated controls. The mean and standard errors were calculated, and significant differences analyzed using one-way ANOVA with Bonferroni’s multiple comparison post-hoc test, where *p* < 0.05 was considered significant.

## Results

### Basal Alox15 Protein Expression in Primary Murine Cortical Neurons and Undifferentiated SH-SY5Y Cells

**Fig. 1:**
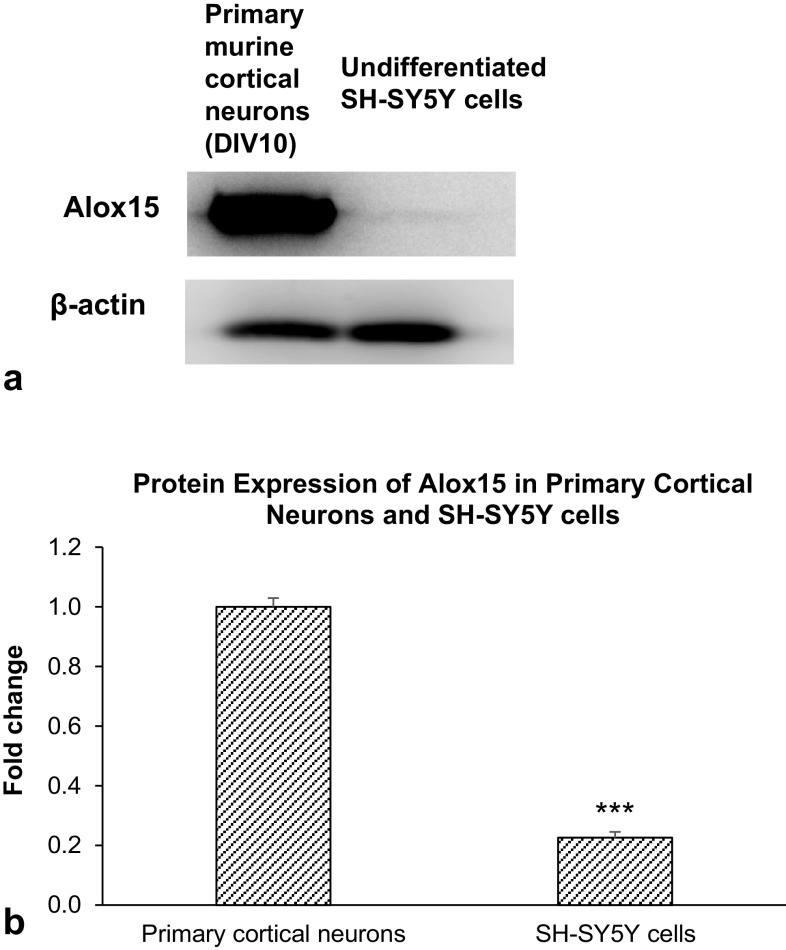
Western blot analyses. **a** Immunoblot of Alox15 protein expression in primary murine cortical neurons and undifferentiated SH-SY5Y neuroblastoma cells at 10 days-in-vitro. **b** Densitometric analysis of protein band intensity normalized to β-actin. Data were analyzed by Student’s *t* test. The mean and standard error are indicated. n = 3 in each group. Asterisk indicates significant difference between undifferentiated SH-SY5Y neuroblastoma cells and primary cortical neurons at 10 days-in-vitro, **P < 0.001

Western blot analyses revealed that Alox15 protein was highly expressed in primary murine cortical neurons at day 10 in vitro. In comparison, undifferentiated SH-SY5Y cells expressed very low levels of Alox15 protein (Fig. 1a, b).

### Effect of Alox15 on Primary Murine Neurons at 3, 5 and 10 Days-In-Vitro (DIV)

**Fig. 2:**
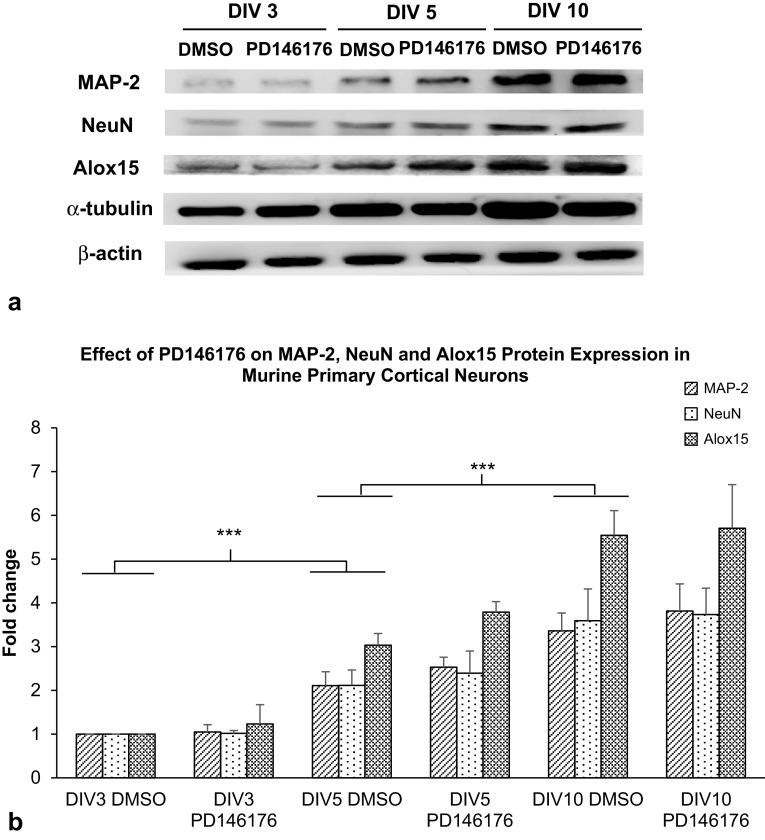
Effect of Alox15-specific inhibitor PD146176 on primary murine neuronal markers and Alox15 expression during different stages of neuronal development, from DIV3 to DIV10. **a** Immunoblot of MAP-2, NeuN and Alox15 protein expression in primary cortical neurons of different days in culture. **b** Densitometric analysis of protein band intensity normalized to β-actin. Data were analyzed by one-way ANOVA with post-hoc Fisher’s protected *t* test. The mean and standard error are indicated. n = 3 in each group. Asterisk indicates significant differences, ***P < 0.001

There was an increase in neuronal markers MAP-2, NeuN and α-tubulin with time in culture, consistent with predicted maturation down a neuronal lineage (Fig. 2a). Interestingly, there was also a large increase in Alox15 protein expression with time, consistent with the notion that Alox15 expression increases in mature neurons. Treatment of primary murine neurons with PD146176 did not affect the protein expression of MAP-2, NeuN, α-tubulin or Alox15 itself, indicating the activity of Alox15 does not have a direct effect on maturation of neurons (Fig. 2b).

### Effect of HDAC and HAT Inhibitors on Alox15 mRNA Expression in Primary Murine Cortical Neurons at 10 Days-In-Vitro

No significant differences in Alox15 mRNA and protein expression were found after treatment of primary murine cortical neurons with 0.5 µM TSA nor co-treatment of TSA with 20 µM C646 or NU9056 (data not shown).

### Effect of HDAC Inhibitors on Alox15 mRNA Expression in Undifferentiated SH-SY5Y Cells

Significant increases in Alox15 mRNA expression were found after treatment with 0.5 µM TSA (*p* < 0.001), 5 mM sodium butyrate (*p* < 0.001), 5 µM MS-275 (*p* < 0.001) and 10 nM depsipeptide (*p* < 0.001), compared to vehicle controls (Table 1). In contrast, treatment with TMP 195 showed no increase in Alox15 mRNA expression (Table 1). A small but significant increase in Alox15 mRNA expression (*p* < 0.05) was detected after treatment with 15 µM tubacin, compared to vehicle controls (Table 1).

**Table 1:** Real-time RT-PCR results of Alox15 mRNA expression following treatment of undifferentiated SH-SY5Y cells with various HDAC inhibitors

Treatment of undifferentiated SH-SY5Y cells with	Alox15 mRNA expression with respect to (wrt) DMSO control (fold change)	*p* value wrt DMSO control
TSA (0.5 µM)	1435 ± 415	< 0.001
NaBT (5 mM)	990 ± 119	< 0.001
MS-275 (5 µM)	1127 ± 87	< 0.001
Depsipeptide (10 nM)	539 ± 38	< 0.001
TMP195 (1 µM)	1.6 ± 0.7	> 0.05
Tubacin (15 µM)	3.2 ± 0.5	< 0.05

Data were analyzed by one-way ANOVA with Bonferroni’s multiple comparison post-hoc test. The mean and standard error are indicated. n = 4 in each group

### Effect of HAT Inhibitors and TSA on Alox15 Expression in Undifferentiated SH-SY5Y Cells

**Fig. 3:**
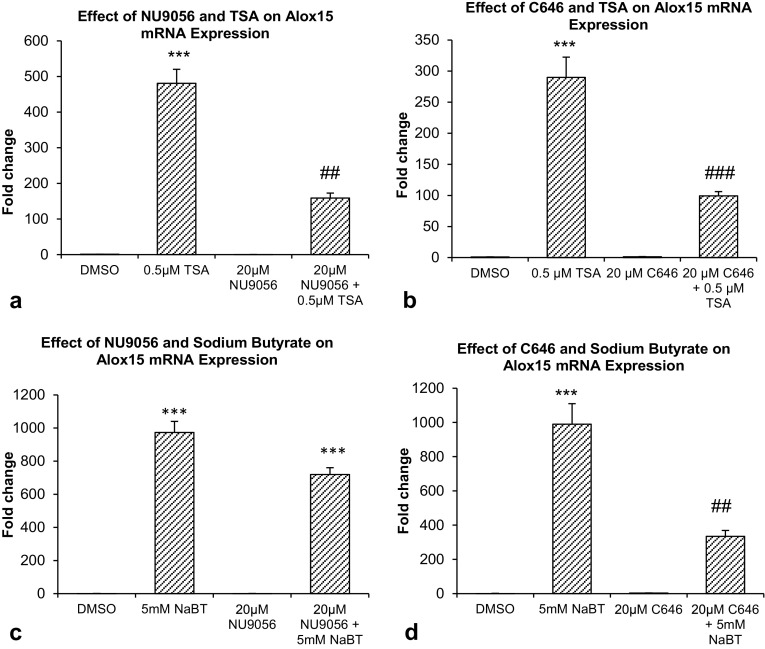
Real-time RT-PCR results. **a** Effect of NU9056 and TSA on Alox15 mRNA expression. **b** Effect of C646 and TSA on Alox15 mRNA expression. **c** Effect of MB-3 and TSA on Alox15 mRNA expression. **d** Effect of NU9056 and sodium butyrate (NaBT) on Alox15 mRNA expression. **e** Effect of C646 and NaBT on Alox15 mRNA expression. Data were analyzed by one-way ANOVA with Bonferroni’s multiple comparison post-hoc test. The mean and standard error are indicated. n = 4 in each group. Asterisk indicates significant difference compared to vehicle-treated group, ***P < 0.001. Number sign indicates significant difference compared to 0.5 μM TSA, ^##^*P* < 0.01, ^###^*P* < 0.001

Significant increase in Alox15 mRNA expression was found after treatment with TSA. Co-treatment of 20 μM NU9056 and TSA suppressed the TSA-induced increase in mRNA expression by 67.0% (*p* < 0.01) (Fig. 3a). Co-treatment with 20 µM C646 suppressed the effect of TSA treatment on Alox15 mRNA expression by 65.7% (*p* = 0.001) (Fig. 3b), but co-treatment with 20 µM of the GCN5-specific HAT inhibitor MB-3 did not reduce the TSA-induced increase in Alox15 mRNA (data not shown).

**Fig. 4:**
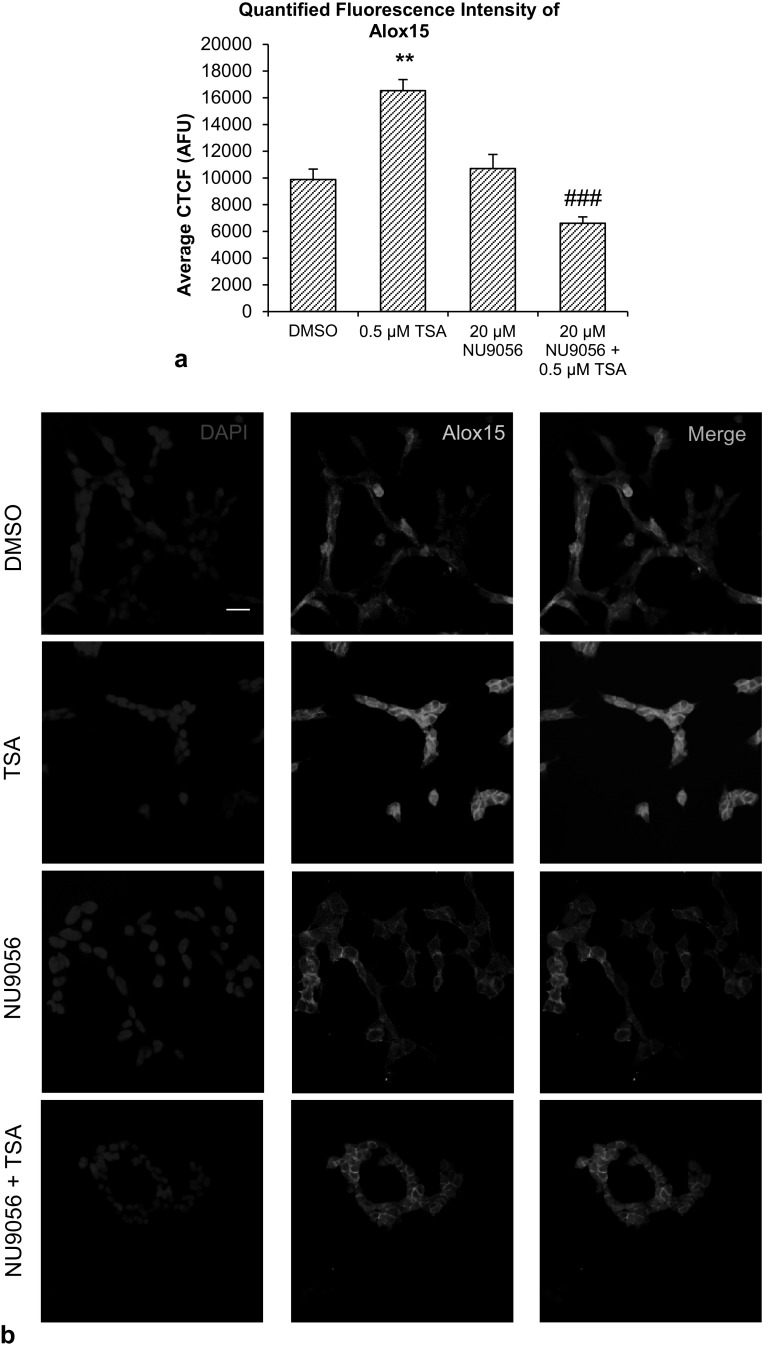
Effect of Tip60-specific HAT inhibitor, NU90546, and TSA. **a** Quantification of ten high-power microscopic fields from four biological replicates per group, analyzed and computed as average corrected total cell fluorescence (CTCF) values. **b** Immunofluorescence images of SH-SY5Y cells expressing Alox15 protein after NU9056 and TSA treatment. Scale bar = 20 µm. Data were analyzed by one-way ANOVA with Bonferroni’s multiple comparison post-hoc test. The mean and standard error are indicated. n = 4 in each group. Asterisk indicates significant difference compared to vehicle-treated group, *P < 0.05, **P < 0.01 and ***P < 0.001. Number sign indicates significant difference compared to 0.5 μM TSA, ^##^*P* < 0.01, ^###^*P* < 0.001

**Fig. 5:**
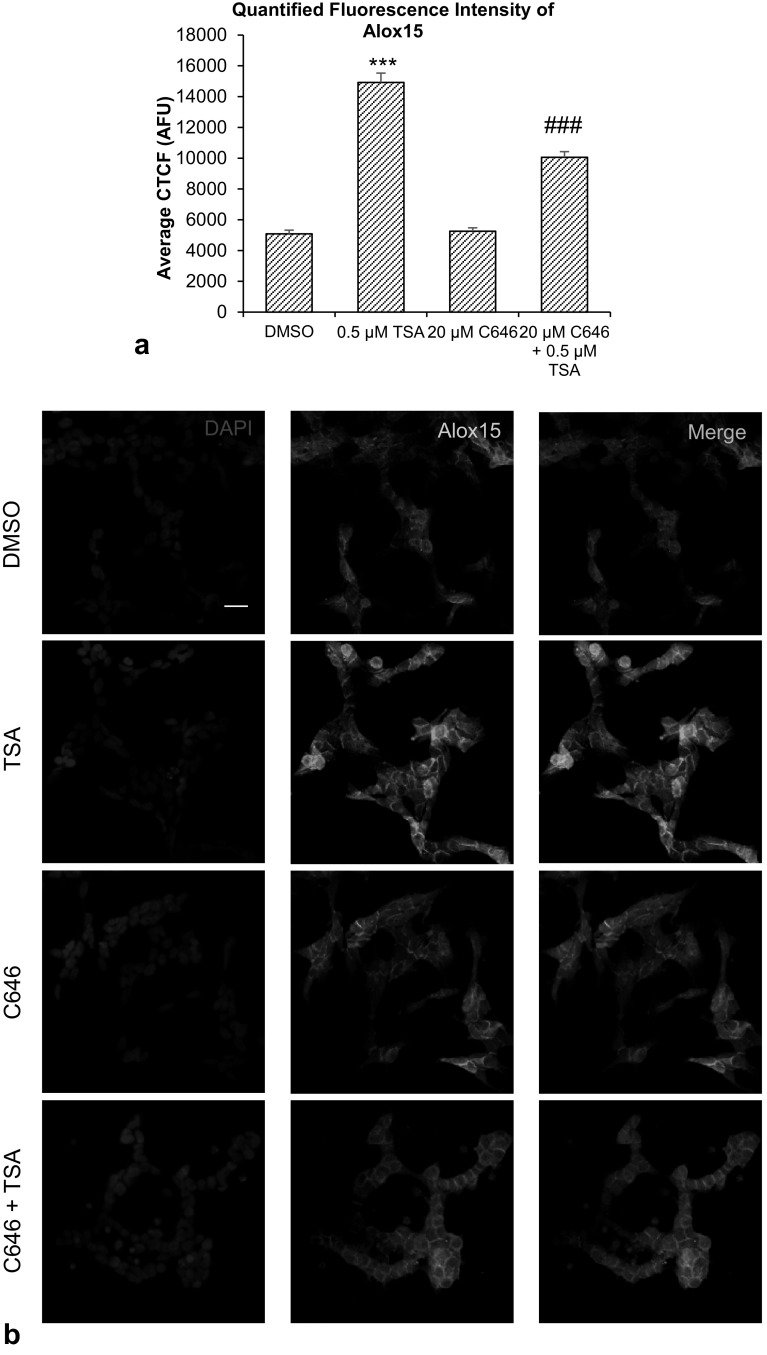
Effect of p300-specific HAT inhibitor, C646, and TSA. **a** Quantification of ten high-power microscopic fields from four biological replicates per group, analyzed and computed as mean corrected total cell fluorescence (CTCF) values. **b** Immunofluorescence images of SH-SY5Y cells expressing Alox15 protein after C646 and TSA treatment. Scale bar = 20 µm. Data were analyzed by one-way ANOVA with Bonferroni’s multiple comparison post-hoc test. The mean and standard error are indicated. n = 4 in each group. Asterisk indicates significant difference compared to vehicle-treated group, ***P < 0.001. Number sign indicates significant difference compared to 0.5 μM TSA, ^##^*P* < 0.01, ^###^*P* < 0.001

Alox15 immunofluorescence labeling showed localization of Alox15 protein in the cytoplasm. A significant increase in fluorescence intensity was observed after TSA treatment, compared to vehicle controls. Co-treatment of 20 µM NU0956 and 0.5 µM TSA reduced the TSA-induced increase in immunofluorescence (Fig. 4a, b). Co-treatment of 20 µM C646 and 0.5 µM TSA also reduced the TSA-induced increase in immunofluorescence (Fig. 5a, b).

### Effect of HAT Inhibitors and Sodium Butyrate on Alox15 Expression in Undifferentiated SH-SY5Y Cells

Significant increase in Alox15 mRNA expression was found after treatment with sodium butyrate. Co-treatment of 20 μM NU9056 and sodium butyrate did not reduce the sodium butyrate-induced increase in Alox15 mRNA expression (Fig. 3c). In contrast, co-treatment with 20 μM C646 reduced the sodium butyrate-induced increase in Alox15 mRNA expression by 66.2% (*p* = 0.004) (Fig. 3d).

**Fig. 6:**
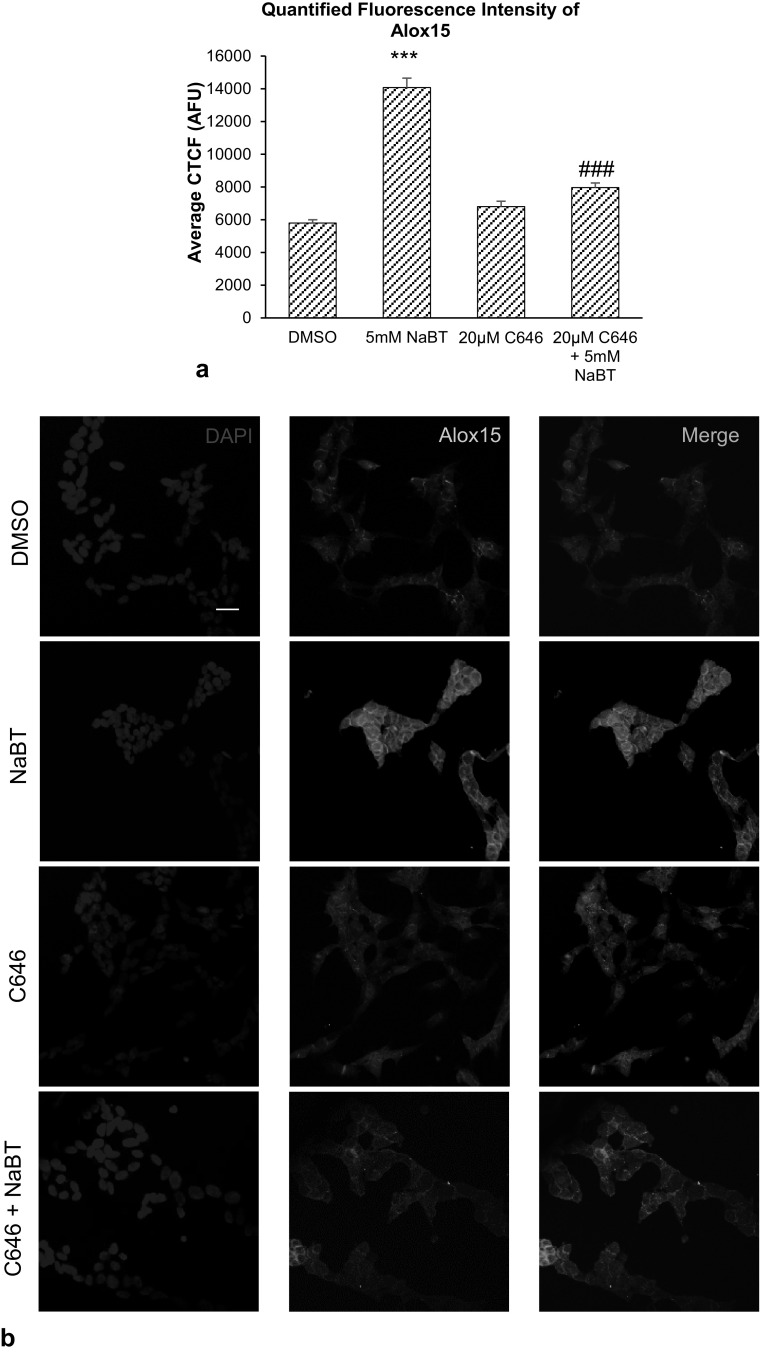
Effect of p300-specific HAT inhibitor C646 and sodium butyrate (NaBT). **a** Quantification of ten high-power microscopic fields from four biological replicates per group, analyzed and computed as average corrected total cell fluorescence (CTCF) values. **b** Immunofluorescence images of SH-SY5Y cells expressing Alox15 protein after C646 and sodium butyrate treatment. Scale bar = 20 µm. Data were analyzed by one-way ANOVA with Bonferroni’s multiple comparison post-hoc test. The mean and standard error are indicated. n = 4 in each group. Asterisk indicates significant difference compared to vehicle-treated group, ***P < 0.001. Number sign indicates significant difference compared to 5 mM sodium butyrate, ^##^*P* < 0.01, ^###^*P* < 0.001

Significant increase in Alox15 immunofluorescence intensity was observed in cells after sodium butyrate treatment, compared to vehicle controls. Co-treatment of 20 µM C646 and 5 mM sodium butyrate reduced the sodium butyrate-induced increase in immunofluorescence (*p* < 0.001) (Fig. 6a, b).

### Effect of HAT Inhibitors and MS-275 on Alox15 Expression in Undifferentiated SH-SY5Y Cells

**Fig. 7:**
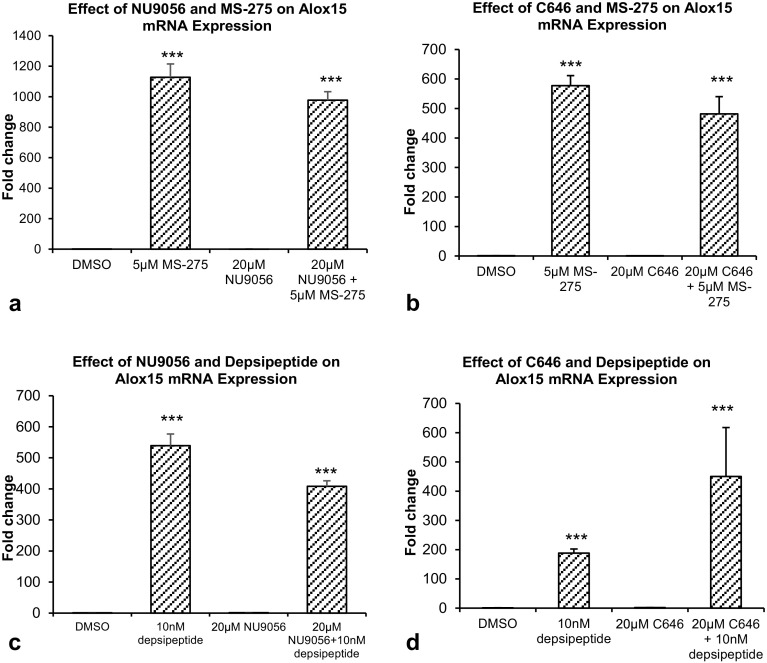
Real-time RT-PCR results. **a** Effect of Tip60-specific HAT inhibitor NU9056 and MS-275 on Alox15 mRNA expression. **b** Effect of p300-specific HAT inhibitor C646 and MS-275 on Alox15 mRNA expression. **c** Effect of Tip60-specific HAT inhibitor NU9056 and depsipeptide, on Alox15 mRNA expression. **d** Effect of p300-specific HAT inhibitor C646 and depsipeptide on Alox15 mRNA expression. Data were analyzed by one-way ANOVA with Bonferroni’s multiple comparison post-hoc test. The mean and standard error are indicated. n = 4 in each group. Asterisk indicates significant difference compared to vehicle-treated group, ***P < 0.001

A significant 1127.2-fold increase in Alox15 mRNA expression was found after treatment with 5 μM MS-275 (*p* < 0.001). Co-treatment of 20 μM NU9056 and MS-275 did not reduce the MS-275 induced increase in Alox15 mRNA expression (Fig. 7a).

In another experiment, a significant increase of 577.4-fold in Alox15 mRNA expression was observed after treatment with 5 μM MS-275 (*p* < 0.001). Co-treatment with 20 μM C646 failed to suppress the MS-275 induced increase in Alox15 mRNA expression (Fig. 7b).

### Effect of HAT Inhibitors and Depsipeptide on Alox15 Expression in Undifferentiated SH-SY5Y Cells

A significant increase of 539.3-fold in Alox15 mRNA expression was found after treatment with 10 nM depsipeptide (*p* < 0.001). Co-treatment with 20 μM NU9056 did not reduce the depsipeptide-induced increase in Alox15 mRNA expression (Fig. 7c).

Likewise, 10 nM depsipeptide significantly increased Alox15 mRNA expression (*p* < 0.001), and co-treatment with 20 μM C646 failed to suppress the depsipeptide-induced increase in Alox15 mRNA expression (Fig. 7d).

### Effect of Alox15 Inhibitor and TSA on Undifferentiated SH-SY5Y Cell Viability

**Fig. 8:**
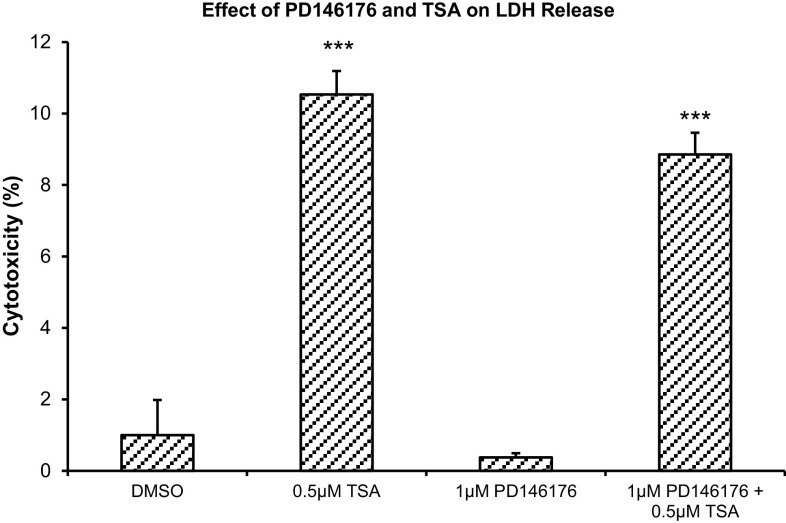
Effect of Alox15-specific inhibitor PD146176 and TSA on LDH release. Data were analyzed by one-way ANOVA with Bonferroni’s multiple comparison post-hoc test. The mean and standard error are indicated. n = 4 in each group. Asterisk indicates significant difference compared to vehicle-treated group, ***P < 0.001

Treatment of SH-SY5Y cells with 0.5 µM TSA caused a significant 10.5-fold in cytotoxicity (*p* < 0.001). Co-treatment with the specific Alox15 inhibitor PD146176 at a concentration of 1 µM did not significantly reduce the cytotoxicity (Fig. 8).

### Effect of RA on SH-SY5Y Cell Morphology, Mean Neurite Length and Neuronal Marker mRNA Expression

**Fig. 9:**
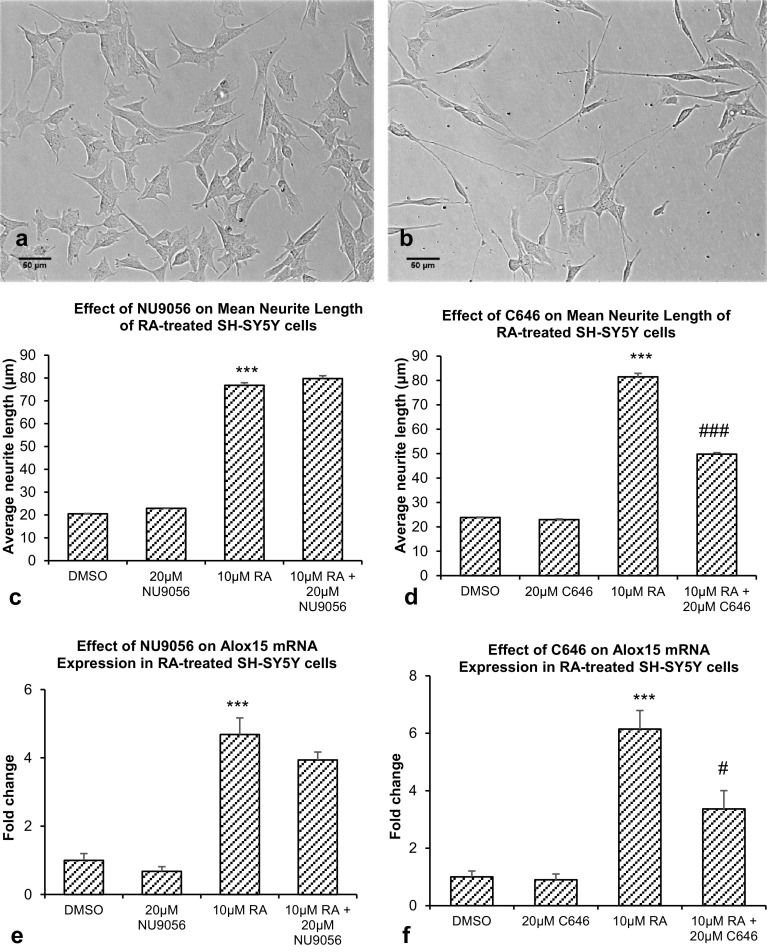
**a, b** Effect of RA on SH-SY5Y cell morphology. **a** Undifferentiated SH-SY5Y cells. **b** SH-SY5Y cells after treatment with 10 μM RA. Scale bar = 50 μm. **c** Effect of RA and NU9056 on average neurite length of SH-SY5Y cells. **d** Effect of RA and C646 on average neurite length of SH-SY5Y cells. **e** Effect of RA and NU9056 on Alox15 mRNA expression. **f** Effect of RA and C646 on Alox15 mRNA expression. Data were analyzed by one-way ANOVA with Bonferroni’s multiple comparison post-hoc test. The mean and standard error are indicated. n = 4 in each group. Asterisk indicates significant difference compared to vehicle-treated group, ***P < 0.001. Number sign indicates significant difference compared to group treated with 10 μM RA, ^###^*P* < 0.001, ^#^*P* < 0.05

Undifferentiated SH-SY5Y cells showed compact morphologies with few neurites from the cell bodies (Fig. 9a). Treatment of these cells with 10 μM RA resulted in increased extension of neurites from cell bodies and reduction in proliferation (Fig. 9b). A significant 2.05-fold increase in neuronal marker NSE mRNA expression was observed after treatment with 10 μM RA, compared with vehicle control (*p* < 0.001) (Table 2). No significant increase in βIII-tubulin mRNA expression was observed after treatment with 10 μM RA, compared with vehicle control (Table 2).

**Table 2:** Effect of RA and TSA on NSE and βIII-tubulin mRNA expression in SH-SY5Y cells

Treatment of undifferentiated SH-SY5Y cells with	Fold change in NSE mRNA expression wrt DMSO control	Fold change in βIII-tubulin mRNA expression wrt DMSO control	Statistical significance of fold change wrt	
			*DMSO*	*RA*
TSA (0.5 µM)	6.4 ± 1.1	10.2 ± 1.7	*p* < 0.001	–
RA (10 µM)	2.1 ± 0.1 (a)	1.3 ± 0.1 (b)	(a) *p* < 0.001	–
			(b) *p* > 0.05	–
RA (10 µM) + TSA (0.5 µM)	9.3 ± 1.0	6.5 ± 0.5	*p* < 0.001	*p* < 0.001

Data were analyzed by one-way ANOVA with Bonferroni’s multiple comparison post-hoc test. The mean and standard error are indicated. n = 4 in each group

Significant increase in mean neurite length of SH-SY5Y cells was observed after treatment with 10 μM RA (73.8 ± 1.2 μm) compared to undifferentiated SH-SY5Y cells (22.2 ± 0.2 μm, *p* < 0.001) (Table 3). Interestingly, a significant 2.9-fold increase in Alox15 mRNA expression was also observed after treatment with 10 μM RA, compared to vehicle control (*p* < 0.01) (Table 3).

**Table 3:** Effect of RA on mean neurite length and Alox15 mRNA expression in SH-SY5Y cells

	Treatment of SH-SY5Y cells with		*p* value wrt DMSO control
	DMSO	RA (10 µM)	
Mean neurite length (µm)	22.2 ± 0.2	73.8 ± 1.2	< 0.001
Alox15 mRNA expression (fold change)	1	2.9 ± 0.7	< 0.01

All data were analyzed by one-way ANOVA with Bonferroni’s multiple comparison post-hoc test, representing mean and standard error of at least four biological replicates

### Effect of RA and HAT Inhibitor on Mean Neurite Length and Alox15 mRNA Expression in SH-SY5Y Cells

Significant increase in mean neurite length of SH-SY5Y cells was observed after treatment with 10 μM RA, where mean neurite length was 76.8 ± 1.2 μm, as compared to undifferentiated SH-SY5Y cells where mean neurite length was 20.5 ± 0.1 μm (*p* < 0.001) (Fig. 9c). Co-treatment with 10 μM RA and 20 μM NU9056 did not result in significant changes in the increase in mean neurite length (Fig. 9c).

In another experiment, significant increase in mean neurite length of SH-SY5Y cells was observed after treatment with 10 μM RA, where mean neurite length was 81.5 ± 1.4 μm, as compared to undifferentiated SH-SY5Y cells where mean neurite length was 23.8 ± 0.2 μm (*p* < 0.001) (Fig. 9d). Co-treatment with 10 μM RA and 20 μM C646 significantly reduced the mean neurite length by 38.9% to 49.8 ± 0.6 µm (*p* < 0.001) (Fig. 9d).

Significant increase of 4.7-fold in Alox15 mRNA expression was found after treatment with 10 μM RA (*p* < 0.001) (Fig. 9e). Co-treatment with 10 μM RA and 20 μM NU9056 did not result in significant reduction in Alox15 mRNA expression (Fig. 9e).

In another experiment, significant increase of 6.1-fold in Alox15 mRNA expression was observed after treatment with 10 μM RA (*p* < 0.001) (Fig. 9f). Co-treatment with 10 μM RA and 20 μM C646 significantly reduced Alox15 mRNA expression by 45.3% (*p* < 0.05) (Fig. 9f).

## Discussion

The present study was carried out to elucidate the effect of histone acetylation on Alox15 expression in SH-SY5Y cells, which were originally derived from a bone tumor [39]. The current literature provides evidence that undifferentiated SH-SY5Y cells continuously proliferate, express immature neuronal markers, and lack mature neuronal markers [40]. Undifferentiated SH-SY5Y cells are considered to have the properties of immature catecholaminergic neurons. In comparison, fully differentiated SH-SY5Y cells express a variety of neuronal markers such as growth-associated protein (GAP-43), NeuN, synaptophysin (SYN), synaptic vesicle protein II (SV2), NSE and MAP-2; but not glial markers such as glial fibrillary acidic protein (GFAP). In further support that differentiated SH-SY5Y cells have many of the characteristics of neurons, removal of BDNF results in cellular apoptosis. This suggests that survival of differentiated SH-SY5Y cells is dependent on trophic factors, similar to mature neurons [41]. Alox15 mRNA expression was significantly increased after treatment with general HDAC inhibitors TSA and sodium butyrate and the Class I HDAC inhibitors MS-275 and depsipeptide, in undifferentiated SH-SY5Y cells. Results indicate a role of class I HDACs such as HDACs 1, 2, 3 and 8 [42] in regulating Alox15 expression in these cells. In comparison, no significant effect was observed after treatment with the class IIa-specific HDAC inhibitor TMP 195, suggesting that class IIa HDACs are not involved. The class IIb HDAC6 inhibitor tubacin did not upregulate Alox15 mRNA expression, as HDAC6 deacetylates tubulin but not histones [43]. Our observations are consistent with previous studies which showed HDAC inhibitor-mediated transcriptional upregulation of Alox15 in colon [23, 24, 44], pancreatic [45] and breast [46] cancer cells. Global H3 and H4 acetylation [24], as well as demethylation of the CpG islands on the 5′ promoter region [47], have been shown to upregulate Alox15 gene expression in colon cancer cells. HDAC inhibitors may work with transcription factors [48] or synergistically with cytokines [49] to regulate gene expression.

A balance between acetylation and deacetylation of histone proteins is important in regulating gene expression [26], and we therefore elucidated a possible role of HATs in regulating Alox15 expression. Treatment with the p300 HAT inhibitor C646 together with the general HDAC inhibitors TSA or sodium butyrate, showed that p300 HAT is regulating Alox15 expression in SH-SY5Y cells. This is consistent with a previous study, which reported a role of p300 HAT in Alox15 expression in colon cancer cells [24]. p300 HAT and its associated protein CREB-binding protein (CBP) interacts with STAT6 [50], and acetylation of histones by p300 HAT allows IL-4-induced-STAT6 to bind to the promoter, facilitating gene transcription of Alox15 [49]. Treatment with NU9056, a specific Tip60 HAT inhibitor reduced the increase in Alox15 mRNA and protein expression induced by TSA, but NU9056 failed to suppress the increase in Alox15 mRNA expression induced by sodium butyrate. The GCN5 HAT inhibitor MB-3 did not inhibit the increase in Alox15 mRNA expression by TSA, suggesting that the GCN5 HAT is not involved in Alox15 gene regulation (data not shown). Results indicate the importance of p300 HAT, and perhaps Tip60 HAT, in increasing Alox15 expression. Both p300 and Tip60 HAT inhibitors, however, failed to suppress increases in Alox15 mRNA expression that are induced by MS-275 or depsipeptide. The reason for this is unknown, but could be due to histone acetylations that are increased by MS-275 and depsipeptide induced HDAC inhibition, but unaffected by p300 or Tip60 HAT inhibition. Treatment with TSA caused a small amount of cytotoxicity in SH-SY5Y cells as demonstrated by LDH released. This is consistent with previous findings that TSA can be detrimental to neuronal cell viability [51]. Co-treatment with the Alox15 inhibitor PD146176 did not reduce cell death induced by TSA, suggesting that Alox15 is not involved in cell damage under these conditions.

Differentiated SH-SY5Y cells possess neuron-like morphology and express neuronal markers, and are more similar to neurons than undifferentiated cells [52]. Treatment of SH-SY5Y cells with 10 μM RA resulted in upregulation of the neuronal marker NSE, which is consistent with a previous report [53]. Retinoic acid-induced differentiation of SH-SY5Y cells has been shown to lead to changes in acetylation status of histone H3 of genes [54], and in this study, we found significant upregulation of Alox15 mRNA expression after treatment with 10 μM RA. Co-treatment with C646 but not NU9056 suppressed the increase in Alox15 expression, indicating a role of p300 HAT in retinoic-differentiation induced Alox15 upregulation. Increasing Alox15 expression was found in murine primary neurons from 3 days to 10 days-in-vitro, reaching high levels of expression by 10 days-in-vitro; and HDAC inhibitors had no effect on increasing Alox15 expression in these neurons at this time. Inhibition of Alox15 did not affect the expression of MAP-2, NeuN, α-tubulin or Alox15 itself. Together, results indicate regulation of Alox15 mRNA expression in neuroblastoma cells by HDACs and HAT, and increasing levels of Alox15 expression with differentiation.

The prenatal or developing brain is highly sensitive to environmental influences and epigenetic modifications, which can affect gene expression in immature neurons [55]. In contrast, the mature brain is less sensitive to such effects [56]. In view of our recent findings of a role of Alox15 in synaptic plasticity [15] and this study, we postulate that one such environmental influence that can affect learning and memory in the developing brain, may take the form of epigenetic effects on Alox15 and metabolites of DHA. Further work is necessary to elucidate the detailed mechanisms of effect of histone acetylation on Alox15 expression.
